# Layered double hydroxide nanoparticles as an adjuvant for inactivated foot-and-mouth disease vaccine in pigs

**DOI:** 10.1186/s12917-020-02689-6

**Published:** 2020-12-04

**Authors:** Peng Wu, Yunfeng Zhang, Xinyue Yin, Yanhua He, Qian Zhang, Chuangfu Chen

**Affiliations:** 1 College of Animal Science and Technology, Shihezi University, Xinjiang, China; 2 College of Life Technology, Shihezi University, Xinjiang, China; 3 State Key Laboratory of Sheep Genetic Improvement and Healthy Production/ Xinjiang Academy of Agricultural and Reclamation Sciences, Xinjiang, China

**Keywords:** LDHs, FMDV, NPs, Adjuvant, Immune response

## Abstract

**Background:**

Foot-and-mouth disease (FMD) is a highly transmissible disease that leads to vast economic losses in many countries. Prevention using inactivated vaccines is one effective measure used to control FMD. Unfortunately, inactivated FMD vaccines provide only short-term protection and require a cold-chain system. In recent years, many studies have shown that layered double metal hydroxides (LDHs) carrying antigens can be used to strongly induce immune responses. In this study, LDH nanoparticles (NPs) were prepared by hydrothermal synthesis. LDH particle size, electric potential, and morphology were measured and observed. The adsorption capacity of LDH NPs to FMDV was tested. The effects of LDH as an adjuvant on inactivated FMDV vaccines were further evaluated and compared with commercial FMDV Montanide ISA-206 in BALB/C female mice and Yorkshire pigs.

**Results:**

LDH NPs were successfully prepared with a uniform particle size of ~ 87.21 nm, regular edges, a loose hexagonal shape and positive zeta charge of 32 mV. The maximum absorption concentration was 0.16–0.31 μg FMDV/μg LDH. In the mouse experiment, antibody levels in group LDH + FMDV were significantly higher compared to group saline + FMDV (*P* < 0.01) from days 42–98 and were significantly higher to group ISA-206 + FMDV on day 56 post-immunization (*P* < 0.05). After day 14 post-immunization, IFN-γ content was significantly increased (*P* < 0.05). In the pig experiment, antibody levels in both the ISA-206 + FMDV and LDH + FMDV were positive and were significantly higher compared with the PBS group on day 7 (*P* < 0.005). Antibody levels in 90% pigs were positive on day 56 in the LDH group. The neutralizing antibody levels in the LDH and ISA-206 groups were significantly higher from days 7–28 compared to the PBS control group (*P* < 0.05). Thus, LDH NPs were effective at inducing an immune response against FMDV.

**Conclusions:**

LDHs with a loose hexagonal shape and a positive charge were prepared and evaluated as adjuvant for FMD vaccine. It was demonstrated that LDHs can induce immune responses in mice and pigs. In addition, the LDHs produced antibodies continuously which may indicate a slow-release effect. The study shows that LDHs may act as a potentially useful FMDV adjuvant.

## Background

Foot-and-mouth disease (FMD) is a highly contagious disease in cloven-hoofed animals, which spreads rapidly [1]. The disease affects many areas of the world, often causing extensive epizootics in livestock, particularly farm cattle and swine, although sheep, goats and many wild species are also susceptible [1, 2]. High morbidity, a complex host-range and broad genetic diversity make FMD prevention and control exceptionally challenging [2]. In most countries, susceptible livestock are immunized with inactivated foot-and-mouth disease virus (FMDV) vaccine in order to control the disease. Finding truly safe and effective vaccines, especially those that induce cell-mediated immunity, is the key to prevent and control the disease.

Adjuvant development plays a major role in vaccine technology. The reasonable use of adjuvants in vaccines not only lessens the use of antigens, but also stimulates the immune system quickly and enhances the immune response. The choice of adjuvant is particularly important. Several kinds of vaccine adjuvants have been studied for their potency to promote immune responses to FMDV vaccines. These adjuvants include mineral oil (ISA-206 and ISA-201) [3], saponins (Quil-A) [4], Toll-like receptor (TLR) ligands (targeting pattern recognition receptors) [5, 6], cytokines (IFN-a, IFN-g, IL-1, IL-2, IL-15, IL-18 and GM-CSF) [7–9] and liposomes [10]. Currently the commercial FMDV adjuvants used include mineral oil-based adjuvants such as Montanide ISA-206, ISA-201, and aluminum hydroxide. Oil emulsions mainly rely on the strong reactogenicity to induce immunoreactions, which normally trigger severe side effects including hemolysis, swelling or necrosis at the injection site [11]. Aluminum hydroxide is approved by the FDA for use in humans because of its safety and efficacy [12]. Nevertheless, Aluminum hydroxide typically induces a classical antibody-mediated (Th2) response rather than cell-mediated (Th1) immunity, and therefore is not suitable for vaccination against diseases such as intracellular infections [13]. The FDA-approved adjuvant also has undesirable features, it is non-biodegradable and consequently remains in situ longer than 1 year [14]. Aluminum hydroxide also frequently produces a strong inflammatory reaction at the injection site [15]. Although some new adjuvants have been developed in recent years, but excellent adjuvant with good safety, efficacy, targeting, stability, controllable release, highly efficient immunity and low cost may be some of the key research directions in the future.

Nanoparticles (NPs) and nanomaterials show great potential as next-generation adjuvants with desirable physicochemical features and reduced undesirable drawbacks and side effects [16]. To date, NPs such as mesoporous silica NPs [17], chitosan NPs [18], gold NPs [19], poly (D,L-lactic-co-glycolic acid) (PLGA) NPs [20], clay nanomaterials (i.e. layered double hydroxide and hectorite) [21, 22] have proven their capacity to boost immune responses as effective adjuvants.

Layered double hydroxide (LDH) is hydrotalcite-like clay, represented by the chemical formula [M^2+^ _1-x_M^3+^_x_ (OH)_2_]^X+^+[A^n-^]_x/n_·mH_2_O [23]. LDHs are formed by weathering of basalt in the nature. LDH is a layered structure: the laminates have a structural positive charge, and the interlayers are composed of anions and water molecules. The interlayers are bound together by electrostatic interaction. Recent results have demonstrated that dispersion-stable LDH NPs are efficient vaccine carriers, stimulate higher levels of antibodies for a longer period, maturate dendritic cells (DCs) and promote stronger specific T cell immune responses [24]. For example, antigen BSA-Cy7 loaded LDH complexes generate loosely structured agglomerates either in solution or within nodules formed at the injection site and recruit immune cells into injection nodules and over a prolonged period [25]. LDH-adjuvanted multiple-antigen vaccine formulations can efficiently stimulate strong humoral, cellular and mucosal immune responses that are capable of preventing *E. coli* from adhering to mammalian cells more efficiently than the commercial adjuvant formulation [21, 26]. A dispersion-stable LDH-based vaccine induced stronger cytotoxic T-lymphocyte (CTL) responses and significantly inhibited tumor growth [27]. Therefore, LDH is a promising adjuvant for vaccine due to the small particle size, stable dispersion, large specific surface area, positive charge, large cargo load, sustained release, easy absorption, low toxicity, low cost, and significantly improved cellular immune response.

Adjuvant plays a major role in the FMD vaccines as the vaccine comprises of inactivated FMDV antigen. Currently, Montanide ISA-206 as a commercial FMDV adjuvant induces relatively strong immune response, but has shortcomings due to its side effects at the injection site. Therefore, it is imperative to develop novel adjuvants for FMD vaccine. In this study we attempted to investigate the efficacy of LDH NPs as an adjuvant for inactivated FMDV vaccines in mice and pigs in comparison to Montanide ISA-206. LDH may be an effective and safe adjuvant to improve FMD vaccine efficacy.

## Results

### Physicochemical properties of LDH NPs

**Fig. 1:**
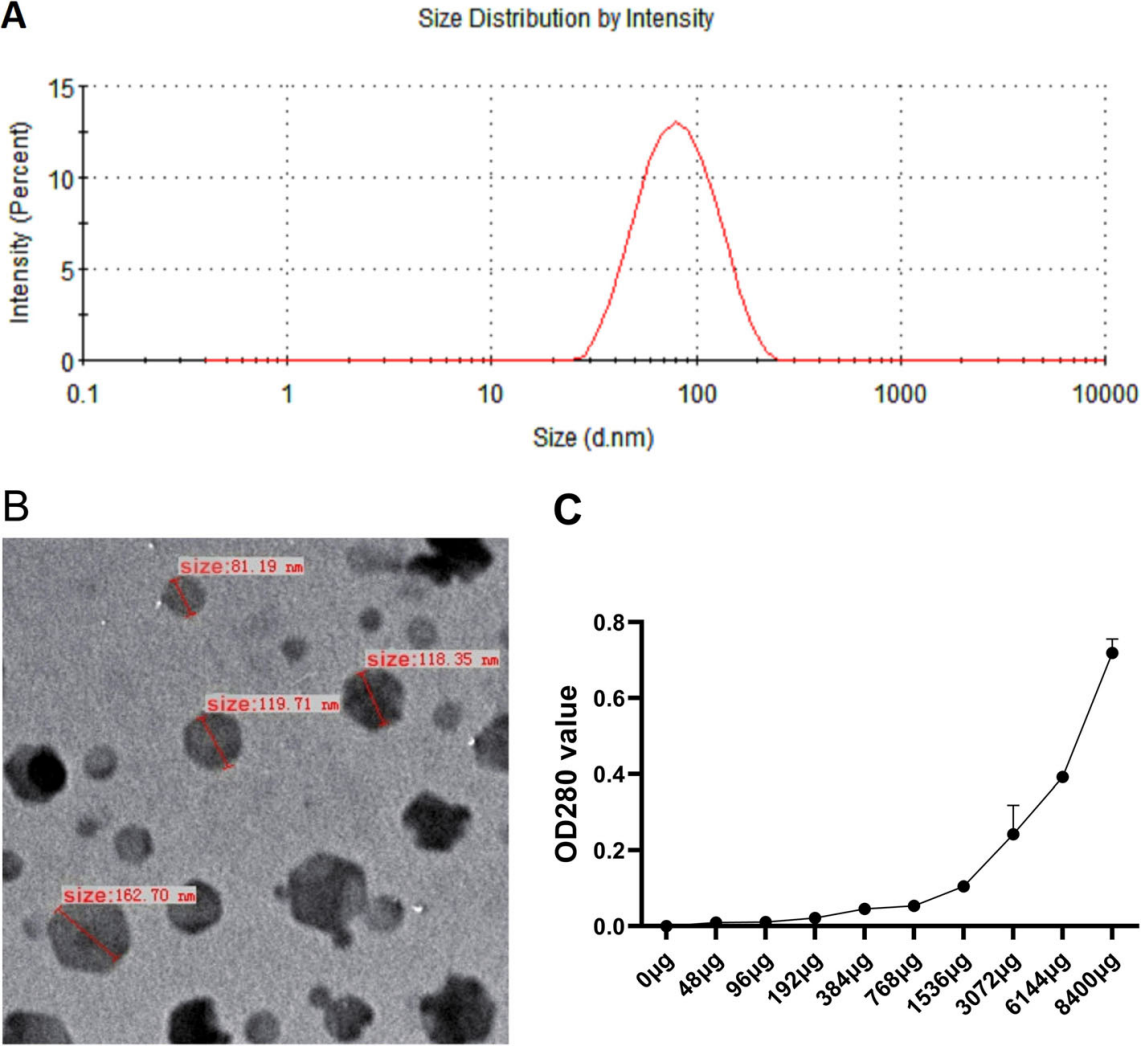
Electron micrograph of LDHs and adsorption capacity of LDHs. **a** Particle size distributions of LDHs with single peak. **b** Representative scanning electron microscope image of LDHs. **c** Absorption of FMDV to LDHs. The X-axis represents the amount of inactivated FMDV in different tubes. The Y-axis values is the A280, which represents the amount of unbound FMDV 146S particle after adsorption with LDHs. Data are expressed as mean ± standard error of the mean (*n* = 2)

LDH NPs (Mg_2_Al-Cl-LDH) were synthesized using rapid precipitation method followed by hydrothermal treatment. The particle size of LDH had only one peak at 87.21 nm, which showed homogeneously dispersed suspension (Fig. 1a). The equivalent mean hydrodynamic diameter was 70.96 nm, and LDH size was no greater than 200 nm. The LDH NPs were positively charged (zeta potential 32 mV). The TEM image showed that the LDH crystallites were well crystallized with a typical hexagonally-shaped morphology (Fig. 1b). The final concentration of LDH was 48.62 mg/ml.

### Effective adsorption of FMDV by LDH NPs

The capacity of LDH NPs to adsorb FMDV 146S was determined by the amount of free virions in the supernatant after the binding of LDHs and FMDV. After 1536 μg inactivated FMDV was added to 100 μl (4862 μg) of LDHs particles, a large number of viruses began to appear in the supernatant as observed by OD280 (Fig. 1c). The LDH NPs were able to absorb the maximum FMDV 768–1536 μg, that is, the maximum absorption concentration was 0.16–0.31 μg FMDV/μg LDH.

### Cytotoxicity of LDH NPs

**Fig. 2:**
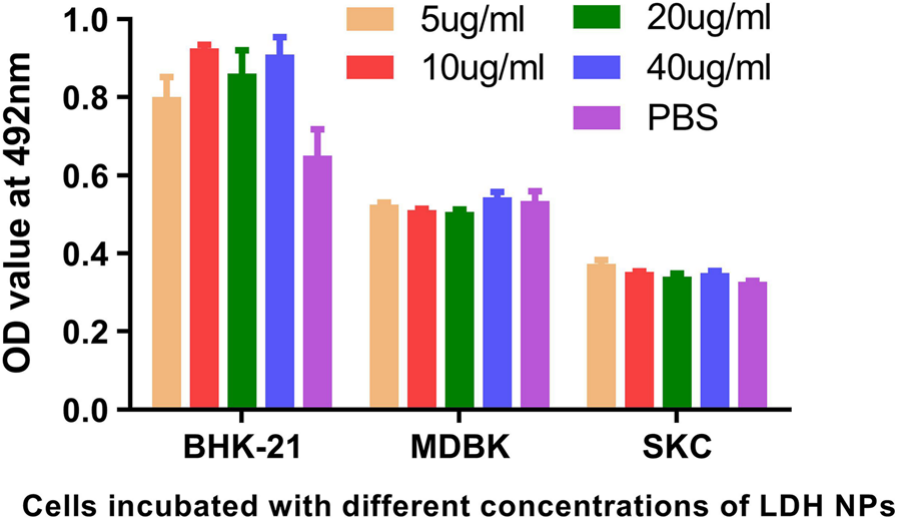
LDH NPs cytotoxicity in BHK-21, MDBK and SKC cells. Data are expressed as mean ± SD (*n* = 3)

LDH toxicity was evaluated using BHK-21, MDBK, and SKC cells. Incubation with different concentrations of LDH for 38 h had no effect on any of the three cell types, and there was no statistically significant difference when compared with the PBS control group (Fig. 2). The results showed that LDH NPs can be used as an adjuvant in animals.

### Evaluation of immune response in LDHs+inactivated virus immunized BALB/C mice

**Fig. 3:**
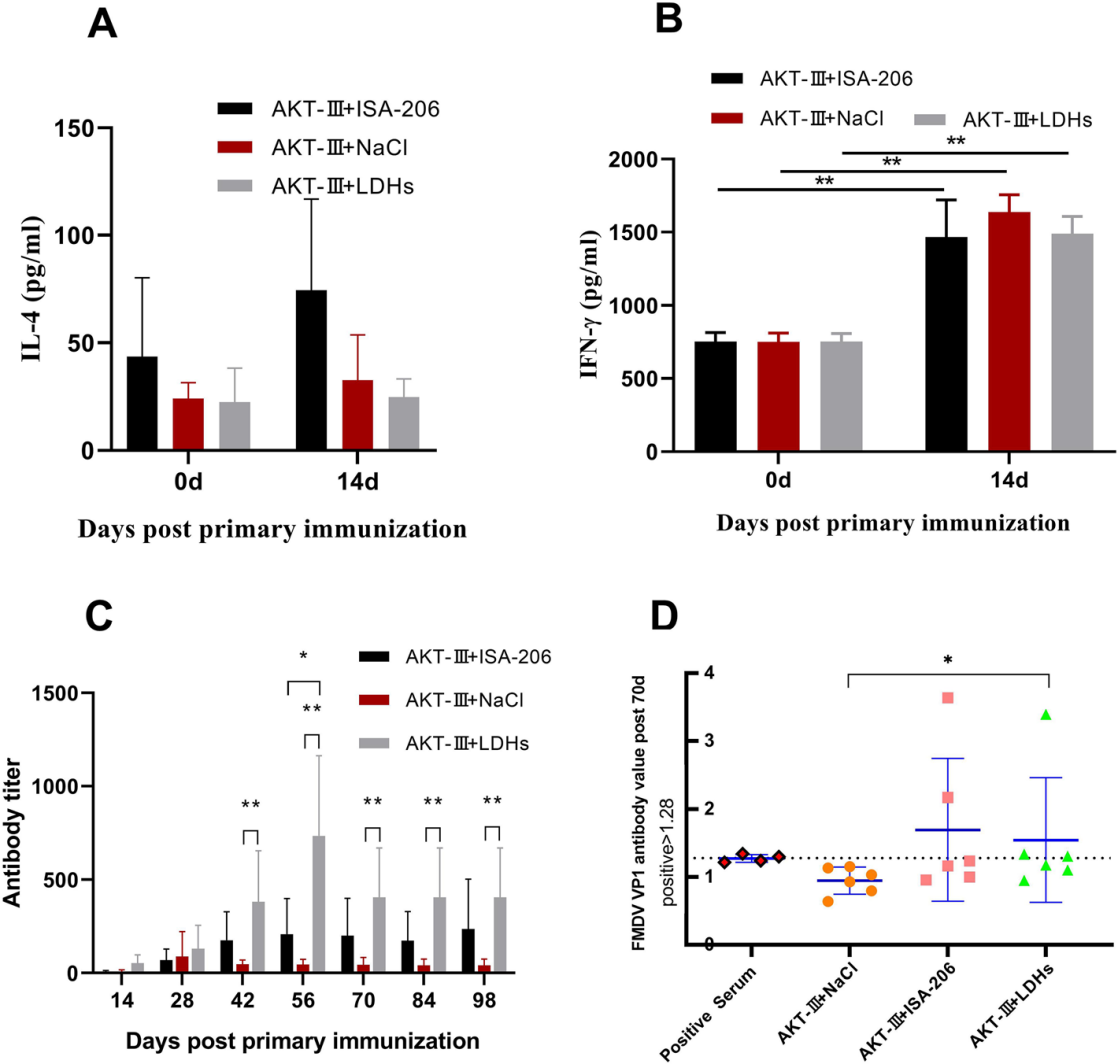
Results from immunized mice. **a** IL-4 levels of immunized mice. **b** IFN-γ levels of immunized mice. **c** FMDV antibody titer of immunized mice at different times by ELISA. **d** FMDV-VP1 antibody levels of immunized mice on the day 70. VP1 specific antibody level in the Y-axis is represented as log10 value of dilution. Data are expressed as mean ± SD (*n* = 6). * *p* < 0.05, ** *p* < 0.01

The LDH was formulated with inactivated FMDV to determine its potential adjuvant efficacy. IL-4 and IFN-γ secretions were measured 14 days post-immunization. With respect to IL-4, there was no difference among the groups (Fig. 3a). As can be seen in Fig. 3b, the IFN-γ level was significantly higher in three groups vaccinated with FMDV+LDHs, FMDV+ISA-206 and FMDV+saline compared to pre-immunization (*P* < 0.01). Th1 and Th2 represent the two arms of the adaptive immune response [28]. In general, IL-4 is secreted by activated Th2 cells, and IFN-γ is secreted by Th1 cells. The results showed that the adjuvant LDH and Montanide ISA-206 caused strong cellular immunity, but the level of humoral immunity was still weak in the early stage. The average antibody titer in different stages was measured and results showed a significantly higher levels in antibodies induced by LDHs+FMDV compared to the saline group from day 42 to 98 (*P* < 0.01) and significantly higher compared to the ISA-206 group on day 56 post-immunization (*P* < 0.05) (Fig. 3c). The humoral immune response reached the highest on day 56 and remained at a high level on days 70, 84, and 98 (Fig. 3c). FMDV VP1 antibody levels on day 70 post-immunization showed that the VP1 antibody titer of the LDH group was significantly higher compared to the control group immunized with virus alone (P < 0.05). In addition, the positive rate in LDH group was 1/2, the ISA-206 group was 1/3, and the virus only group was negative compared with the positive serum (Fig. 3d). The antibody titer of the liquid phase blocking ELISA was basically the same as that of FMDV VP1. The results showed that LDH as an adjuvant not only induced cellular and humoral immunity, but also had sustained release.

### Evaluation of immune response in LDHs+inactivated virus immunized pigs

**Fig. 4:**
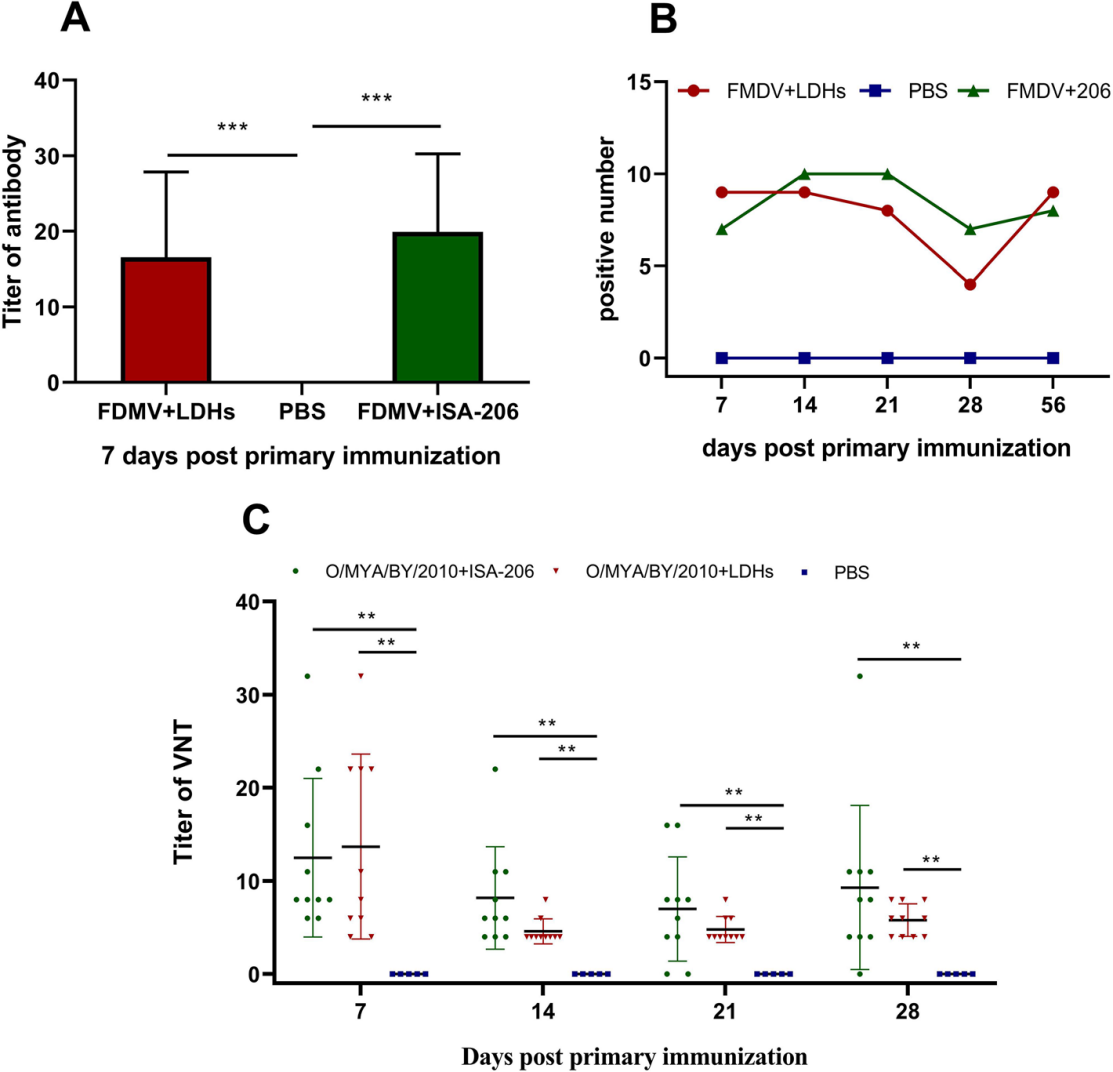
Results of immunized pigs. **a** Antibody level of immunized pig on the day 7 by ELISA. **b** Positive number of pigs at different times as showed by ELISA. The level of antibody titer of pig is above 1:32, which is considered as positive. **c** Titer of VNT at different times. Data are expressed as mean ± SD (*n* = 10). * *p* < 0.05, ** *p *< 0.01, *** *p* < 0.005

To better evaluate the potential efficacy of LDH as an adjuvant, 20 pigs were immunized with LDH NPs + inactivated FMDV O/MYA/BY/2010 or ISA-206 + inactivated FMDV O/MYA/BY/2010. On day 7 post-immunization, antibody levels in both the Montanide ISA-206 adjuvant group and the LDH adjuvant group were positive and were significantly higher (*P* < 0.005) compared to the PBS group, in which antibody level was negative (Fig. 4a). There was no significant difference between the LDH group and the Montanide ISA-206 group. In the LDH group, antibody levels of 9 pigs (10 in total) were positive on day 7. The number of positive pigs decreased on day 28, but returned to 9 on day 56 (Fig. 4b), which may indicate that LDH was continuously releasing antigen. In conclusion, the FMDV antibody level detected by ELISA was not very high, which may have been due to the low dose of antigen used.

Results of the virus micro-neutralization test showed that antibody levels of pigs immunized with LDH + FMDV and ISA-206 + FMDV ranged from 1:4 to 1:32, and were significantly higher from day 7 to day 28 compared to the PBS control group (*P* < 0.01) (Fig. 4c). The pig numbers of the LDH group with positive titers was higher than the ISA-206 adjuvant group on day 28 post-immunization. These results demonstrate that the efficacy of LDH NPs for inducing specific antibody responses against FMDV was comparable to that of commercial Montanide ISA-206 adjuvant in pigs.

## Discussion

LDHs are a large family of two-dimensional (2D) anionic clay materials [23], and have a very strong load capacity [29] and can carry proteins, viruses, DNA and other anions substances in the interlayer galleries. The LDH NPs were able to immobilise a high amount of the three *E.coli* protein antigens, with saturation adsorption amounts of 1.10, 0.28 and 0.42 mg mg^− 1^ intimin β, proprietary antigen 1 and proprietary antigen 2 for LDH [26]. Virions are more complex than proteins, and the saturated absorption of FMDV was 0.16–0.31 μg μg^− 1^ for LDH. The positive charge property can combine with the negative charge group of the cell membrane to enter the cell smoothly [30]. An additional advantage is that virus combined with LDH can be protected compared to conventional adjuvant. LDH has good safety as a drug carrier and vaccine adjuvant. LDH NPs loaded with anti-cancer drug doxorubicin [31] could deliver the drug to the location of the cancer cells and achieve a targeted effect, indicating that the cytotoxicity of the LDH particles was negligible. At the same time, many studies showed that LDH could be used as an adjuvant to induce a high level of immune response in vivo, and showed no toxicity to the body [21, 22, 25, 26]. In the study, LDH toxicity was evaluated using BHK-21, MDBK, and SKC cells, showing that LDHs had very good biocompatibility and had no toxicity to the kidney cells of hamster, cattle and sheep. Therefore, LDH NPs can be used as an adjuvant in animals.

Nano-particulate have the characteristics of large specific surface area, many surface-active centers and high reactivity, which can produce volume effect and surface effect. LDHs are small in diameter and usually similar to pathogens in size. Studies had shown that NPs entering the body can be quickly recognized by antigen presenting cells (APCs) and swallowed up [32]. The use of NPs to deliver soluble antigens significantly increased the uptake efficiency of dendritic cells (DCs) compared with the use of soluble antigens alone, and in some cases increased the uptake efficiency by 30 times [33]. IFN-γ and IL-4 are important immunomodulators that have multiple biological functions. IFN-γ is a Th1 cytokine produced by activated T cells and NK cells, which has a variety of biological activities. IFN-γ is anti-viral, anti-parasitic and inhibits cell proliferation, which can induce at Th1 type immune response [34]. IL-4 is a cytokine produced by activated Th2 cells, which can enhance the interaction between B cells and T cells, promote the humoral immune response, and induce mononuclear-macrophages to express MHC-II molecules [35]. Th1 cells induce cell-mediated immunity whereas Th2 cells induce strong antibody responses [29]. FMDV combined with LDH enhanced the immunogenicity in Fig. 3. IFN-γ content increased significantly in mouse, indicating that LDH induced cellular immune response at an early stage. The average antibody titer maintained at a high level in mouse, showing that LDH induced humoral immune response. The current study also demonstrated that sustained antigen release can be accomplished using an LDH nano-adjuvant, which promotes antigen presentation and produces a long-lasting and efficient memory immune response, thus reducing the number of vaccinations and the amount of antigen [24–27]. The humoral immune effect remained at a high level until 98th day in mouse after a single injection of LDH + virus, indirectly indicating that LDH may have a slow-release effect and continuously produced antibodies. In a word, LDHs has its unique advantages in spatial effect, antigen presentation ability and sustained release ability.

Traditionally, new FMD vaccines/adjuvants are studied in cattle (OIE manual). Among the seven known FMDV serotypes, type A and type O are the most widespread in China, and type Asia I has been successfully controlled [36]. FMDV AKT-III (Serotype A) strain was used locally to prevent foot-and-mouth disease in cattle and sheep. Therefore, inactivated AKT-III was applied in mice to facilitate adjuvant evaluation in cattle at later stage. Pigs pose a challenge in that they tend to respond poorly to many FMD vaccines compared to cattle and sheep [37], and FMDV (Serotype O) often occurs in pigs and are less stable and more prevalent. If the LDH adjuvant works well in pigs, the protective effect will be even better in cattle and sheep. Therefore, it might be more valuable to assess this adjuvant using FMDV O/MYA/BY/2010 (Serotype O) in pigs. In the study, both the ELISA and virus neutralization test showed that LDH NPs had effectively induced specific antibody responses against FMDV in pigs. The antibody levels were slightly different by ELISA and VNT, possibly because the ELISA is more sensitive and detected specific and some non-specific antibodies, while the VNT detected only specific virus neutralizing antibodies.

LDH can potentially be an alternative adjuvant for FMD vaccines due to its many advantages. For example, the preparation process of LDH is relatively simple, and provides a basis for large-scale industrial production. Due to the low production cost of LDH, the use of it as an adjuvant will greatly reduce the breeding cost to farmers. As an inorganic substance, with stable dispersion properties, LDHs can be stored for a long time. Because of the production and preparation through high temperature and high pressure, the product is sterile, which is a necessary condition for the production of vaccines. This was a preliminary study and more work is required before LDHs can be recommended for wider application as vaccine adjuvant. Moreover, the vaccination trials with virus challenge studies should be carried out in more number of animals including cattle.

## Conclusion

LDH NPs could be synthesized using hydrothermal treatment and the particle size, electric potential, and morphology of LDHs showed good characteristics. In order to verify the effectiveness of LDH as an adjuvant to FMDV vaccine, mice and pigs were immunized with FMDV antigen with either LDH or the commercial adjuvant Montanide ISA-206. LDH induced immune responses in both mice and pigs, and produced antibodies continuously which may indicate a slow-release effect, demonstrating the potential of LDH NPs as a useful nano-adjuvant for FMDV. As the next step, LDH as an adjuvant should also be studied in other susceptible hosts including cattle, sheep and goats, so that LDH can be used in production practice as soon as possible.

## Methods

### Materials

The FMDV AKT-III (Serotype A) and O/MYA/BY/2010(Serotype O) inactivated virus and Montanide ISA-206 adjuvant were provided by Tiankang Biotechnology (Urumqi, China). The FMD antibody ELISA test kits were purchased from the Lanzhou Veterinary Research Institute of the Chinese Academy of Agricultural Sciences (Lanzhou, China). Mouse IFN-γ ELISA kit (SEKM-0031) and IL-4 detection kit (SEKM0005) were purchased from Solarbio Science & Technology (Beijing, China). RecombiVirus FMDV VP1 (Serotypes O + A + A1) IgG ELISA Kit (Cat. # RV-400750-1, 96 tests) was purchased from Alpha Diagnostic International Inc. (San Antonio, USA). The MTS Cell Proliferation Colorimetric Assay Kit (Catalog # K300–500) was purchased from BioVision (Milpitas, USA). Fetal bovine serum and DMEM medium were purchased from Gibco. MgCl_2_.6H_2_O, AlCl_3_.6H_2_O, and NaOH were purchased from Sigma.

### Cells and animals

Baby hamster kidney cells (BHK-21 cells) and Madin-Darby bovine kidney cells (MDBK cells) were purchased from Cell Resource Center, IBMS, CAMS/PUMC (Beijing, China). Sheep kidney cells (SKCs) and mouse macrophages were provided by Xinjiang Key Laboratory of Local and Ethnic Diseases, Shihezi University (Shihezi, China). The 2-months-old BALB/C female mice weighed ~ 20 g. The 3-month-old Yorkshire pigs weighed ~ 20 kg. The mice and pigs were healthy and had not been used for the other experiments. The mice and pigs were provided by Huaxing Laboratory Animal Farm (Zhengzhou, China).

### Preparation and characterization of LDH nanoparticle suspension

Mg_2_Al-Cl-LDH NPs were prepared by rapid precipitation and subsequent hydrothermal treatment [28, 38]. After mixing 0.7 M MgCl_2_ solution with 0.3 M AlCl_3_ solution uniformly, 0.45 M NaOH solution was quickly added, stirred for 10 min, and put in the reaction kettle at 100 °C for 16 h. The chemical formula of LDH is Mg_1.9_Al (OH) _5.8_ (1/2CO^2−^ _3_, Cl)_1.0_·5H_2_O. The particle size and potential of the LDHs samples were analyzed using the Malvern particle size analyzer. Scanning electron microscope (SEM) was used to observe the morphology of the nanometer adjuvant, the acceleration voltage was 80 kV, and the magnification rate was 30,000 × .

### FMDV adsorption by LDH NPs

The 146S concentration was estimated by sucrose density gradient method followed by spectrophotometry at 259 nm wavelength. To quantify the adsorption of FMDV on LDH NPs, 100 μl LDH adjuvant (48.62 mg/ml) was added to each EP tube, and FMDV O/MYA/BY/2010 146S (6 mg/ml) was added by volume 0 μl, 8 μl, 16 μl, 32 μl, 64 μl, 128 μl, 256 μl, 512 μl, 1024 μl, and 1400 μl. Deionized water was then added to a volume of 1500 μl per tub. After shaking the mixture for 10 min, the nanoparticle/virus complexes were recovered by centrifugation at 5000 rpm for 20 min and the amount of unbound FMDV in the supernatant was estimated via nanodrop at 280 nm (A280).

### Cytotoxicity test [39]

BHK-21, MDBK, and SKC preserved in liquid nitrogen were revived with warm water at 37 °C, and cell culture fluid (90% DMEM + 10% FBS) was added. A total of 6 × 10^3^ cells were transferred into 96-well plates and incubated for 3 h. Triplicate wells were treated with LDH at final concentrations of 5 μg/ml, 10 μg/ml, 20 μg/ml, and 40 μg/ml. The plates were incubated at 37 °C in 5% CO_2_ for 38 h, and 20 μl/well MTS reagent was then added and wells were incubated for 3 h at 37 °C. The absorbance was measured at 492 nm after shaking the plate.

### Immunization of pigs with LDHs and inactivated virus

All mouse experiments were performed according to the guidelines of the Animal Ethics Committee of the Shihezi University. Specific pathogen free BALB/C mice were kept in a cage and sterilized wood dust as the bedding material. The mice were allowed free access to clean water and food. The ambient temperature was ~ 27 °C. After the experiment, the mice were euthanized by intraperitoneal injection of excessive sodium pentobarbital (200 mg/kg weight). The mice were randomly divided into three groups, as follows: 125 μl inactivated FMDV AKT-III + 125 μl saline (control group, *n* = 6), 125 μl inactivated FMDV AKT-III + 125 μl ISA-206 adjuvant (n = 6), 125 μl inactivated FMDV AKT-III + 125 μl LDH adjuvant (n = 6). The final antigen concentration was 7.08 μg/ml all three groups. Before injection, inactivated FMDV was mixed 1:1 with commercial Montanide ISA-206 adjuvant and stirred at 600 rpm for 10 min. The other two groups were mixed uniformly and injected. Six female mice in each group were injected subcutaneously. Blood was collected from submandibular vein under abdominal anesthesia with sodium pentobarbital (40 mg/kg). Blood samples were collected on days 0, 14, 28, 42, 56, 70, 84, and 98 and the serum collected. Antibodies were detected using the Lanzhou Veterinary Research Institute liquid phase blocking kit according to the instructions. FMDV VP1 antibody levels were measured on day 70 post-immunization using the RecombiVirus FMDV VP1 (Serotypes O + A + A1) IgG ELISA kit. Secretion levels of IL-4 and IFN-γ were detected using the Solarbio mouse IL-4/IFN-γ assay kit according to the manufacturer’s protocol.

### Immunization of pigs with LDHs and inactivated virus

All pig experiments were performed according to the guidelines of the Animal Ethics Committee of the Shihezi University. The pigs were clean animal (CL) grade and were farmed on the ground with sufficient light. The pigs were allowed free access to clean water and complete diet pellets. All treatments were aseptic and clean feeding. The breeding temperature was ~ 27 °C. After the experiment, the pigs were euthanized by intraperitoneal injection of excessive sodium pentobarbital (200 mg/kg weight). All pigs were randomly divided into groups. Five pigs were raised together without bedding. The PBS control group (*n* = 5) was injected with 1 ml PBS/pigs. The ISA-206 group (*n* = 10) was injected with 0.5 ml inactivated virus O/MYA/BY/2010 + 0.5 ml ISA-206 adjuvant /pigs. The LDH group (n = 10) was injected with 0.5 ml inactivated virus O/MYA/BY/2010 + 0.5 ml LDH NPs /pigs. The final O/MYA/BY/2010 virus concentration in immunized groups was 6 μg/ml. All injections were administered intramuscularly. Blood was collected from anterior vena cava in pigs under abdominal anesthesia with sodium pentobarbital (40 mg/kg). Day 0 prior to immunization and subsequently at day 14, 21 and 28 for serum collection. The FMD antibody level was detected using the Lanzhou Veterinary Institute FMDV type O liquid phase blocking diagnostic kit, and the cut-off titer was greater than 1:32, which was positive. The virus micro-neutralization test (VNT) was performed by Tiankang Biotechnology Co., Ltd. in P3 laboratory.

### Virus micro-neutralization test (VNT) procedure

The titer of neutralizing antibodies was determined by VNT using FMDV O/MYA/BY/2010 with known titre. Convalescence pig serum was used as the standard positive serum. Pig serum without FMD antibodies was used as the negative serum. Maintenance medium (50 μl/well) was added to the 96-well plate. The inactivated sera were diluted two times in series and 50 μl/well was added per each dilution. To the serially diluted sera, 100 TCID_50_ virus in 50 μl was added and incubated for 1 h at 37 °C in 5% CO_2_. Then the BHK21 cell suspension was added and incubated. The cytopathic effect was observed under a microscope after 48 h. Antibody titres are expressed as the final dilution of serum where 50% of wells are protected.

### Data statistics and analysis

Data are presented as mean ± standard deviation (SD). Statistical analysis was performed using GraphPad Prism 8 software, and significant differences were analyzed by Mann-Whitney U test (* *P* < 0.05, ** *P* < 0.01, ***P < 0.005).

## Data Availability

The datasets used and analyzed during the current study are available from the corresponding author on reasonable request.

## Abbreviations

LDHs: Layered double metal hydroxides
FMDV: Foot-and-mouth disease virus
NPs: Nanoparticles
BHK-21: Hamster kidney cells
MDBK: Bovine kidney cells
SKC: Sheep kidney cells
VNT: Virus micro-neutralization test
OD: Optical density
TLR: Toll-like receptor
GM-CSF: Granulocyte-macrophage colony-stimulating factor
CTL: Cytotoxic T-lymphocyte
